# Supplementary material for: Clinical and Oncological Outcomes Following Percutaneous Cryoablation vs. Partial Nephrectomy for Clinical T1 Renal Tumours: Systematic Review and Meta-Analysis
Source: Cancers (Basel). 2024 Mar 17;16(6):1175. doi: 10.3390/cancers16061175 (PMC10968956; doi:10.3390/cancers16061175)
Supplement: Supplementary file 1 [file cancers-16-01175-s001.zip › Supplementary Table S1.pdf]

Author	Anaesthesia type	PCA guidance	PCA system	Additional procedures
Aikawa et al. [11]	Local	CT or MRI	CryoHit system (Galil Medical, Yokneam, Israel) with 17-gauge needles	Transcatheter arterial embolization using iodized oil was routinely performed 2 or 3 days prior to PCA to improve visualization of the renal mass during the same admission periods.
Andrews et al. [12]	General	CT and/or USG	Perc-24 system (Endocare, Inc, Irvine, California) with 13-gauge needles	Hydro-displacement was used in the anterior tumours that were in proximity to the bowel. Ureteral stent was placed before PCA if the tumour was adjacent to the ureter.
Bhindi et al. [13]	General	CT and/or USG	Perc-24 system (Endocare, Inc, Irvine, California) with 13-gauge needles	Hydro-displacement was used in the anterior tumours that were in proximity to the bowel. Ureteral stent was placed before PCA if the tumour was adjacent to the ureter. Non-routine pre-ablation selective arterial embolization was performed with polyvinyl alcohol particles.
Bianchi et al. [14]	Local	CT and USG	The Precise system (Galil Medical, Aarden Hills, MN, USA) with IceRod needles	NR
Chan et al. [15]	General	CT and/or USG	NR	Cold pyeloperfusion was performed in centrally located tumours. Hydro-displacement was used in the tumours that were in proximity to the bowel.
Duus et al. [16]	Local	CT	IceFx system (Boston Scientific, MN, USA) with 17-gauge or 14-gauge needles	When required, adjacent tissues were protected by hydro-displacement with an 18G percutaneous entry thin wall needle with 2% iodine-based saline solution
Fraisse et al. [17]	Local	CT	CryoHit system (Galil Medical, Yokneam, Israel) with 17-gauge needles	Sterile saline or carbon dioxide was infused if there was a risk of freezing-related injuries to the adjacent tissues.
Junker et al. [18]	Local	CT	IceFx system (Boston Scientific, MN, USA) with 17-gauge or 14-gauge needles	Ureteral stent was placed before PCA if the tumour was adjacent to the ureter. Hydro-displacement was used to protect the vital organs and nerves close to the tumour.
Kawaguchi et al. [19]	Local	CT	CryoHit system (Galil Medical, Yokneam, Israel) with 17-guage needles	Arterial embolization with absolute ethanol and ethiodized oil was performed a day before PCA in patients with large or hyper vascular tumours at a high risk of bleeding,
Mason et al. [20]	General	CT and/or USG	Perc-24 system (Endocare, Inc, Irvine, California) with 13-gauge needles	Hydro-displacement was performed in cases with the adjacent bowel. Pre-ablation selective arterial embolization was performed in tumours ≥5 cm to reduce bleeding risk. Retrograde pyeloperfusion via an externalized ureteric stent was used in cases in need of ureteric protection.
Neves et al. [21]	NR	NR	NR	NR
Rembeyo et al. [22]	Local	CT and USG	CryoHit system (Galil Medical, Yokneam, Israel)	NR
Uemura et al. [23]	Local	CT	CryoHit system (Galil Medical, Yokneam, Israel) with 17-guage needles	NR
Yanagisawa et al. [24]	Local	CT or MRI	CryoHit system (Galil Medical, Yokneam, Israel) with 17-guage needles	Transcatheter arterial embolization using a mixture of absolute ethanol and iodized oil was used 2 or 3 days prior to CT-guided PCA to improve visualization of the renal mass.

Abbreviations: CT = computed tomography; **MRI** = magnetic resonance imaging; **NR** = not reported; **PCA** = percutaneous cryoablation; **USG** = ultrasound
