# Supplementary material for: Clinical and Oncological Outcomes Following Percutaneous Cryoablation vs. Partial Nephrectomy for Clinical T1 Renal Tumours: Systematic Review and Meta-Analysis
Source: Cancers (Basel). 2024 Mar 17;16(6):1175. doi: 10.3390/cancers16061175 (PMC10968956; doi:10.3390/cancers16061175)
Supplement: Supplementary file 1 [file cancers-16-01175-s001.zip › Supplementary Table S2.pdf]

**Outcome**	**Variable**	**Subgroup**	**No. of studies** [reference]	**OR** (95% CI) PCA vs. PN	**P-value**	**Heterogeneity, I^2 (%)**
**Overall complication rates**	Clinical T stage	Only cT1a	2 [13,19]	FE: 0.59 (0.26-1.35)	0.21	0%
		Only cT1b	3 [9,13,20]	FE: 1.18 (0.57-2.45)	0.66	0%
		Mixed (cT1a and cT1b)	4 [11,15,16,22]	RE: 0.50 (0.25-0.90)	0.05*	74%
	Surgical approach	Only RAPN	3 [15,19,20]	FE: 0.70 (0.47-1.04)	0.07	10%
		Only LPN	2 [13,22]	FE: 0.76 (0.39-1.48)	0.41	11%
		Mixed (OPN and/or RAPN and/or LPN)	3 [9,11,16]	RE: 0.48 (0.14-1.61)	0.24	81%
**Major (Clavien-Dindo ≥ 3) complication rates**	Clinical T stage	Only cT1a	2 [13,19]	FE: 0.49 (0.04-5.48)	0.56	NA
		Only cT1b	3 [9,13,20]	FE: 0.75 (0.16-3.46)	0.71	0%
		Mixed (cT1a and cT1b)	6 [11,15,16,17,21,22]	FE: 0.35 (0.18-0.67)	0.002*	0%
	Surgical approach	Only RAPN	5 [15,17,19,20,21]	FE: 0.23 (0.09-0.63)	0.004*	0%
		Only LPN	2 [13,22]	FE: 0.62 (0.17-2.34)	0.48	0%
		Mixed (OPN and/or RAPN and/or LPN)	3 [9,11,16]	FE: 0.59 (0.24-1.44)	0.24	0%

* statistically significant

Abbreviations: **FE =** fixed effect; **NA** = not applicable; **LPN** = laparoscopic partial nephrectomy; **OPN** = open partial nephrectomy; **PCA** = percutaneous cryoablation; **PN** = partial nephrectomy; **RE =** random effect; , **RAPN** = robotic assisted partial
