# Supplementary material for: Clinical and Oncological Outcomes Following Percutaneous Cryoablation vs. Partial Nephrectomy for Clinical T1 Renal Tumours: Systematic Review and Meta-Analysis
Source: Cancers (Basel). 2024 Mar 17;16(6):1175. doi: 10.3390/cancers16061175 (PMC10968956; doi:10.3390/cancers16061175)
Supplement: Supplementary file 1 [file cancers-16-01175-s001.zip › Supplementary Table S3.pdf]

**Outcome**	**Variable**	**Subgroup**	**No. of studies** [reference]	**HR** (95% CI) PCA vs. PN	**P-value**	**Heterogeneity, I^2** (%)
**Local recurrence-free survival**	Clinical T stage	Only cT1a	2 [10,13]	FE: 1.66 (0.63-4.39)	0.31	41%
		Only cT1b	4 [9,10,13,20]	FE: 2.83 (1.57-5.09)	<0.001*	25%
		Mixed (cT1a and cT1b)	6 [11,12,15,17,21,22]	FE: 2.32 (1.40-3.85)	0.001*	39%
	Surgical approach	Only RAPN	4 [15,17,20,21]	FE: 3.52 (1.88-6.56)	<0.001*	0%
		Only LPN	2 [13,22]	FE: 1.27 (0.42-3.82)	0.67	42%
		Mixed (open PN and/or RAPN and/or LPN	4 [9,10,11,12]	RE: 1.97 (0.92-4.22)	0.08	59%
**Metastasis-free survival**	Clinical T stage	Only cT1a	2 [10,13]	FE: 0.26 (0.06-1.19)	0.08	0%
		Only cT1b	3 [9,10,13]	FE: 1.31 (0.55 - 3.12)	0.54	14%
		Mixed (cT1a and cT1b)	1 [11]	NA	NA	NA
**Cancer-specific survival**	Clinical T stage	Only cT1a	2 [10,13]	FE: 0.69 (0.09-5.46)	0.73	0%
		Only cT1b	3 [9,10,13]	FE: 1.18 (0.55-2.51)	0.67	0%
		Mixed (cT1a and cT1b)	1 [11]	NA	NA	NA
**Overall survival**	Clinical T stage	Only cT1a	2 [10,13]	FE: 1.99 (1.48-2.67)	<0.001*	0%
		Only cT1b	3 [9,10,13]	FE: 2.20 (1.41-3.44)	<0.001*	63%
		Mixed (cT1a and cT1b)	1 [11]	NA	NA	NA

* statistically significant

Abbreviations: **FE =** fixed effect; **NA** = not applicable; **LPN** = laparoscopic partial nephrectomy; **OPN** = open partial nephrectomy; **PCA** = percutaneous cryoablation; **PN** = partial nephrectomy; **RE =** random effect; , **RAPN** = robotic assisted partial
