# Supplementary figures and images for: Clinical and Oncological Outcomes Following Percutaneous Cryoablation vs. Partial Nephrectomy for Clinical T1 Renal Tumours: Systematic Review and Meta-Analysis
Source: Cancers (Basel). 2024 Mar 17;16(6):1175. doi: 10.3390/cancers16061175 (PMC10968956; doi:10.3390/cancers16061175)

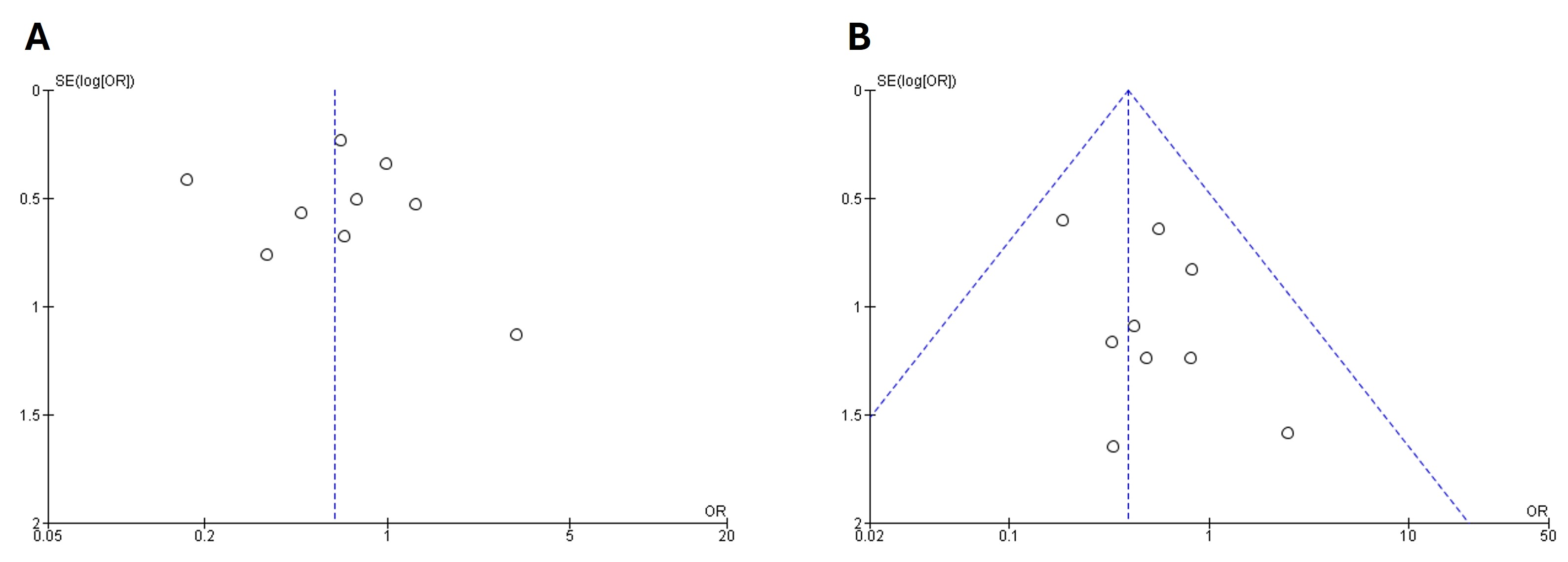

Supplement: Supplementary file 1 [file cancers-16-01175-s001.zip › Supplementary Figure S1.jpg]

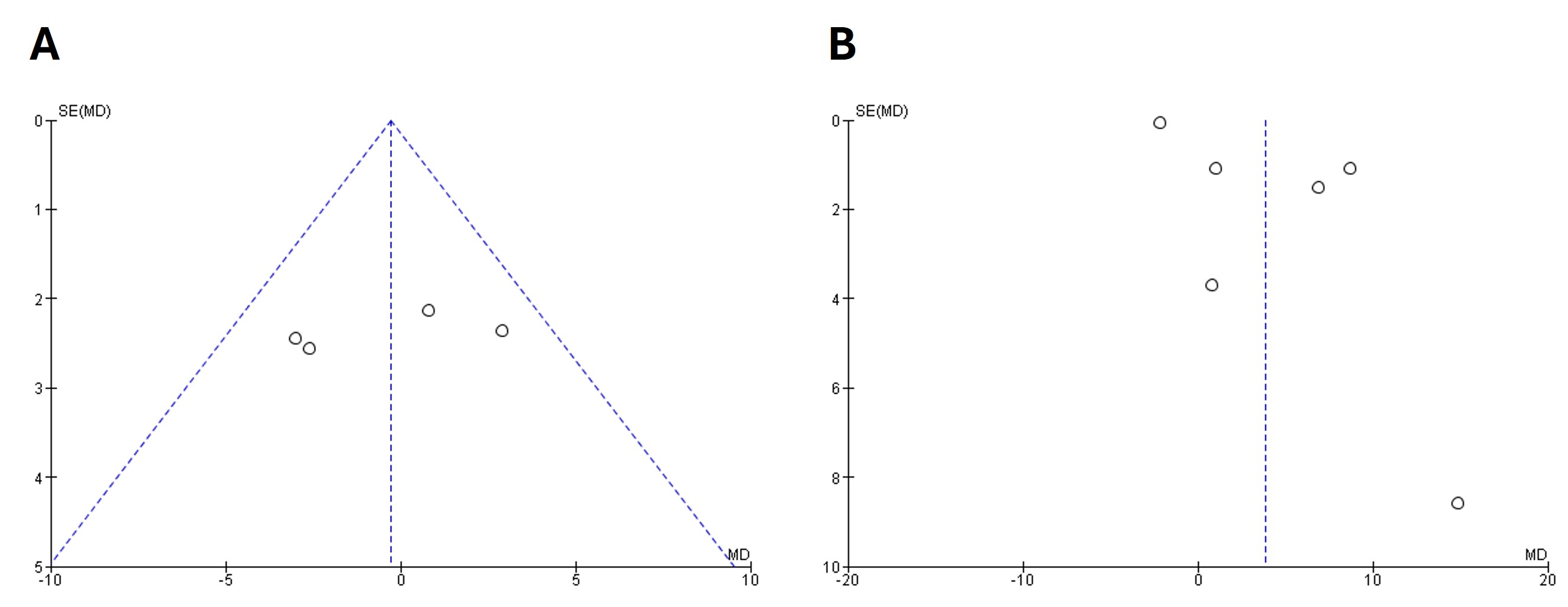

Supplement: Supplementary file 1 [file cancers-16-01175-s001.zip › Supplementary Figure S2.jpg]

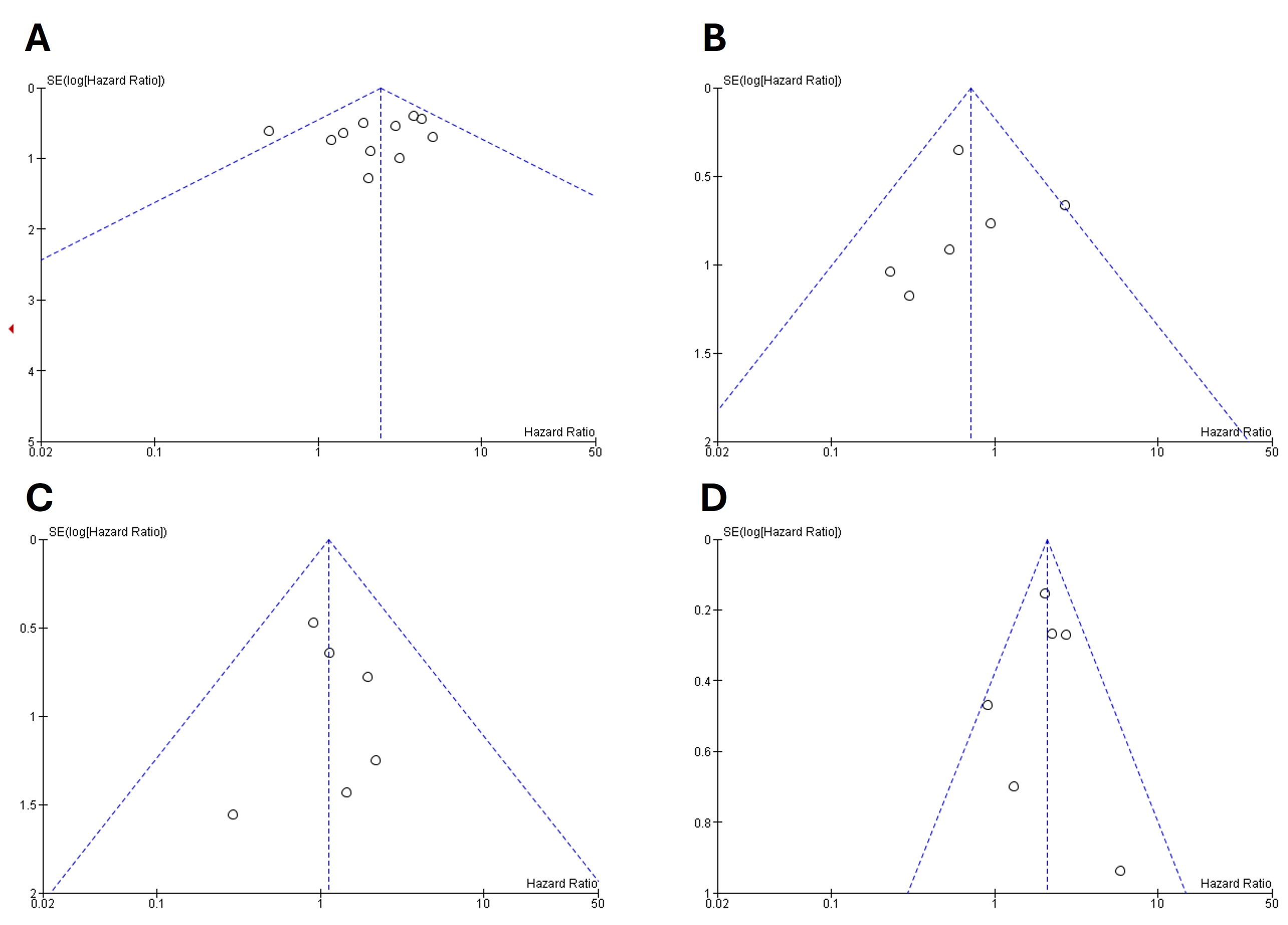

Supplement: Supplementary file 1 [file cancers-16-01175-s001.zip › Supplementary Figure S3.jpg]
